# Role of GABAAR in the Transition From Acute to Chronic Pain and the Analgesic Effect of Electroacupuncture on Hyperalgesic Priming Model Rats

**DOI:** 10.3389/fnins.2021.691455

**Published:** 2021-06-17

**Authors:** Sisi Wang, Junying Du, Danning Xi, Fangbing Shao, Mengting Qiu, Xiaomei Shao, Yi Liang, Boyi Liu, Xiaomin Jin, Jianqiao Fang, Junfan Fang

**Affiliations:** ^1^Key Laboratory of Acupuncture and Neurology of Zhejiang Province, Department of Neurobiology and Acupuncture Research, The Third School of Clinical Medicine, Zhejiang Chinese Medical University, Hangzhou, China; ^2^Department of Anatomy, Cell Biology and Physiology, Stark Neuroscience Research Institute, Indiana University School of Medicine, Indianapolis, IN, United States

**Keywords:** electroacupuncture, hyperalgesic priming, dorsal root ganglions, γ-aminobutyric acid receptor type A, protein kinase C epsilon

## Abstract

Chronic pain is a costly health problem that impairs health-related quality of life when not effectively treated. Regulating the transition from acute to chronic pain is a new therapeutic strategy for chronic pain that presents a major clinical challenge. The underlying mechanisms of pain transition are not entirely understood, and strategies for preventing this transition are lacking. Here, a hyperalgesic priming model was used to study the potential mechanism by which γ-aminobutyric acid receptor type A (GABAAR) in the dorsal root ganglion (DRG) contributes to pain transition. Furthermore, electroacupuncture (EA), a modern method of acupuncture, was administered to regulate pain transition, and the mechanism underlying EA’s regulatory effect was investigated. Hyperalgesic priming was induced by intraplanar injection of carrageenan (Car)/prostaglandin E_2_ (PGE_2_). The decrease in mechanical withdrawal threshold (MWT) induced by PGE_2_ returned to baseline 4 h after injection in NS + PGE_2_ group, and still persisted 24 h after injection in Car + PGE_2_ group. Lower expression of GABAAR in the lumbar DRG was observed in the model rats. Furthermore, activating or blocking GABAAR could reversed the long-lasting hyperalgesia induced by Car/PGE_2_ injection or produced a persistent hyperalgesia. In addition, GABAAR may be involved in Protein Kinase C epsilon (PKCε) activation in the DRG, a mark molecular of pain transition. EA considerably increased the mechanical pain thresholds of hyperalgesic priming model mammals in both the acute and chronic phases. Furthermore, EA upregulated the expression of GABAAR and inhibited the activation of PKCε in the DRG. In addition, peripheral administration of picrotoxin blocked the analgesic effect of EA on the model rats and abolished the regulatory effect of EA on PKCε activation. These findings suggested that GABAAR plays a key role in both the transition from acute to chronic pain and the analgesic effect of EA on hyperalgesic priming.

## Introduction

Chronic pain is a common clinical symptom (Bouhassira et al., 2008; Chen et al., 2016), but the mechanisms underlying this condition remain poorly understood, limiting the ability to adequately treat it. Recent studies have pointed out that the transition from acute to chronic pain involves dramatic changes in the mechanisms of pain (Sun and Chen, 2015; Zha et al., 2015; Price et al., 2018), that not only prolong the duration of pain and greatly aggravate pain but also weaken the effects of treatments. Therefore, elucidating the mechanisms of pain transition is of great significance.

Dorsal root ganglion (DRG) neurons belong to the peripheral sensory ganglion (Matsuka et al., 2020). Numerous primary nociceptive neurons aggregate in the DRG, receive pain signals from the surroundings and play a key role in pain modulation and transmission (Miller et al., 2009; Matsuka et al., 2020). It is known that DRG neurons are responsible for the transition from acute to chronic pain, and the activation of PKCε in DRG neurons plays a key role in hyperalgesic priming type I (Araldi et al., 2019; Wang et al., 2020). However, the other mechanisms underlying pain transition are unknown; specifically, it is unclear whether the inhibitory receptors involved in pain modulation and transmission in the peripheral nervous system contribute to pain transition.

GABA receptor type A (GABAAR) is abundantly expressed in the DRG (Persohn et al., 1991; Ma et al., 1993) and plays a pivotal role in the pain modulation and transmission as an inhibitory receptor (Guo and Hu, 2014; Du et al., 2017). A growing amount of evidence has shown that functional and expressional changes in GABAAR are involved in the generation and maintenance of various chronic pain conditions (Naik et al., 2008; Lee et al., 2018; Lorenzo et al., 2020). Specifically, some reports have indicated that changes in GABAAR function and expression may cause the hyperalgesic priming and that GABAAR can be treated as a potential therapeutic target for hyperalgesia and pain transition in the central nervous system (Kim et al., 2016). However, whether GABAAR is involved in pain transition in the DRG is still unclear.

Electroacupuncture (EA), an effective analgesic method, has been widely used for clinical control of pain and scientific research on this condition (Xiang et al., 2019; Heo et al., 2021). Our previous research has proven that EA can interfere with the transition from acute pain to chronic pain (Wang et al., 2018, 2020). However, the mechanism underlying the effect of EA on the transition from acute to chronic pain is not yet understood clearly. Previous studies have found that EA can not only alleviate chronic neuropathic pain by regulating the expression of GABAAR in the spinal cord (Li et al., 2018; Zheng et al., 2020), but also relieve hyperalgesia in rats with neck incision by upregulating the expression of GABAAR in the DRG (Qiao et al., 2019). These results indicate that GABAAR in the DRG may be an important therapeutic target of the analgesic effect of EA. However, whether EA influences the induction of hyperalgesic priming by regulating GABAAR in the DRG is not known.

In this study, we focused on GABAAR in the DRG and explored its role in pain transition in a model called the hyperalgesic priming model (Reichling and Levine, 2009; Ferrari et al., 2015; Kandasamy and Price, 2015; Yang et al., 2016). Furthermore, we evaluated the treatment effect of EA on hyperalgesic priming model rats and further investigated the potential GABAAR-mediated mechanism underlying EA’s regulatory effect.

## Materials and Methods

### Animals

All experimental procedures were approved by the Animal Care and Welfare Committee of Zhejiang Chinese Medical University, Zhejiang, China (approval no. IACUC-20180319-12). Male Sprague-Dawley rats (weighing 180 to 230 g) were obtained from the Experimental Animal Center of the Shanghai Chinese Academy of Sciences [SCXK (Hu) 2018-0006] and raised in the Experimental Animal Center of Zhejiang Chinese Medical University [SYXK (Zhejiang) 2018-0012]. All rats were fed standard rodent food and housed four per cage (room temperature 23 to 25°C, humidity 55 ± 5%) on a regular light-dark cycle.

### Drug Preparation

Carrageenan (Car) and prostaglandin E_2_ (PGE_2_) were purchased from Sigma-Aldrich (St. Louis, MO, United States). The PKCε agonist ψεRACK (a peptide with the sequence HDAPIGYD and a membrane-permeable sequence) was synthesized by Bankpeptide (Hefei, China). The PKCε inhibitor PKCεV1-2 was purchased from Calbiochem, Millipore Sigma (Darmstadt, Germany). The GABAAR antagonist picrotoxin (A14004), GABAAR agonist muscimol (A13459), and GABABR agonist baclofen (A18248) were purchased from Adooq Bioscience (Irvine, United States). All drugs were dissolved in sterile saline and then diluted to the appropriate concentrations before injection (Car: 1 mg, 100 μL; PGE_2_: 100 ng, 25 μL; ψεRACK: 1 μg, 25 μL; PKCεV1-2: 1 μg, 25 μL; picrotoxin: 1 μM, 25 μL; muscimol: 10 μM, 25 μL; baclofen: 60 μg, 25 μL).

The drugs were administered through intraplantar injection. The injection site was first scrubbed with 75% alcohol.

### Hyperalgesic Priming Model

The hyperalgesic priming model was established by intraplantar injection of 100 μL 1% carrageenan (1st injection) and 100 ng/25 μL PGE_2_ (2nd injection) into the same paw 7 days after the 1st injection.

### Mechanical Withdrawal Threshold

The up-down method was used in this study (Chaplan et al., 1994). Von Frey filaments (Stoelting Co., Thermo, Gilroy, CA, United States) with forces of 0.4, 0.6, 1, 2, 4, 6, 8, 15, and 26 g were used. The rats were placed in a clear plastic cage for 30 min before assessment every day for three continuous days to allow acclimation to the environment. A von Frey filament with a force of 4 g was first applied to the central surface of the hind paw (avoiding the footpad) until the filament bent into an “S” shape, and it was maintained there for 6 s. A filament of a greater or lesser force was then chosen depending on whether the response was negative or positive. The responses were recorded as X or O. The pain threshold was calculated according to the following formula: Mechanical withdrawal threshold (MWT) (g) = [10^(Xf+ κδ)^]/10,000, where “Xf” is the force of the last hair test, the “κ” value is obtained from the k-value table, and “δ” is the average value of the difference between the logarithm of hairs of each force, which is approximately equal to 0.231.

The nociceptive behaviors were quantified during the 8 o’clock to 17 o’clock during the day. And the experimenters were blind to the experimental design.

### EA Administration

All rats in the EA groups were subjected to EA. Four hours after the 1st injection, the EA intervention, which was performed once a day until the end of the experiment, was initiated began after the behavioral test. Needles (0.18 mm × 13 mm) were inserted into the bilateral Zusanli (ST36) and Kunlun (BL60) acupoints at a depth of 5 mm and then connected to a HANS Acupuncture Point Nerve Stimulator (HANS-200A Huawei Co., Ltd., Beijing, China). The stimulation parameters were as follows: 2/100 Hz and an intensity of 0.5 mA, 1.0 mA, and 1.5 mA (the intensity increased every 10 min) for a total of 30 min.

Sham EA was also performed. Needles of the same size were inserted subcutaneously into the animals at the ST36 and BL60 acupoints (at a depth of 1 mm). The needles were connected to the same stimulator, but no electrical stimulation was administered.

### Immunofluorescence

The rats were sacrificed 24 h after the 2nd injection after MWT assessment was completed. The rats were anesthetized with 2% pentobarbital sodium (40 mg/kg, i.p.). Then, the rats were quickly perfused with 0.9% NaCl (4°C) followed by 4% paraformaldehyde in 0.1 M phosphate-buffered saline (PBS) for prefixation. The ipsilateral L4–L6 DRGs were removed and postfixed in 4% paraformaldehyde for 3 h at 4°C before being transferred to 15 and 30% sucrose for dehydration and stored in a −80°C freezer. The DRGs were transversely sectioned (10 μm) with a cryostat and dried at 37°C for 20 min. Then, the sections were blocked with 5% normal donkey serum in TBST (1% Tween-20) for 1 h at 37°C. Then, the sections were incubated with primary antibodies diluted in blocking solution overnight at 4°C. The primary antibodies were mouse anti-GABAARα2 (ab193311, Abcam, Cambridge, United Kingdom) and rabbit anti-PKCε (ab124806, Abcam, Cambridge, United Kingdom). Then, the sections were washed with TBST and incubated with donkey anti-rabbit (Alexa Fluor 488-labeled) or goat anti-mouse (Cy3-labeled) secondary antibody at 37°C for 1 h (diluted with 5% normal donkey serum in TBST). Images were taken using an Imager. M2 microscope (ZEISS, Germany). The analysts were blind to the experiment design.

### Western Blotting

To measure the protein expression of PKCε and GABAAR, the rats were deeply anesthetized as previously described after the last behavioral test. The L4-L6 DRGs were quickly excised and stored at –80°C for further analysis. The tissues were then lysed by adding RIPA lysis buffer (Beyotime, China) containing 1% PMSF (Beyotime, China) and a protease/phosphatase inhibitor cocktail (Applygen, China) to the samples. The target proteins were centrifuged at 14,000 rpm at 4°C for 5 min, and the protein concentration was measured with a BCA protein assay kit. The protein (20 μg) was denatured in loading buffer at 100°C for 10 min, separated on 5/8% SDS-PAGE gels and transferred to polyvinylidene difluoride (PVDF) membranes (Merck KGaA, Darmstadt, Germany). After blocking with 5% skim milk at room temperature for 1 h, the membranes were incubated with rabbit anti-PKCε (ab124806, Abcam, Cambridge, United Kingdom) and mouse anti-GABAARα2 (ab193311, Abcam, Cambridge, United Kingdom) as the primary antibodies and mouse anti-β-actin (HRP-conjugated) (ab20272, Abcam, Cambridge, United Kingdom) as the internal control overnight at 4°C. Then, the membranes were incubated with horseradish peroxidase (HRP)-conjugated goat anti-rabbit IgG (1:5,000, Abcam, United States) or goat anti-mouse (WD0990, Dawen Biotec, Hangzhou) for 1 h at room temperature. The signals were developed using an ECL kit (Pierce, Rockford, IL, United States), and ImageQuant TL 7.0 analysis software (GE, United States) was used to analyze the intensity of the bands. The analysts were blind to the experiment design.

### Experimental Design

In this study, we explored the GABAAR-mediated mechanism underlying EA’s regulatory effect on hyperalgesic priming in three steps.

In step one, the involvement of GABAAR in pain transition in the lumbar DRG was explored. The rats were first randomly assigned to three groups: (1) the Normal group, (2) the sham hyperalgesic priming (NS + PGE_2_) group, and (3) the hyperalgesic priming (Car + PGE_2_) group. MWT changes in hyperalgesic priming rats were investigated. The expression level of GABAAR in the DRG was investigated by immunofluorescence and western blotting.

Then, we first confirmed weather PGE_2_ injection, either after NS or Car injection, changed the function of GABAAR. Muscimol (a selective GABAAR agonist) and picrotoxin (a selective GABAAR antagonist) was used to selectively activate or block GABAAR. To show the PGE_2_ injection will not change the GABAAR function, the rats were randomly divided into 1) the NS + PGE_2_ + NS group and 2) NS + PGE_2_ + Pic^24*h*^ group (administered NS/picrotoxin ipsilaterally 24 h after the 2nd injection before MWT assessment) or (1) the NS + PGE_2_ + NS^24*h*^ group and (2) NS + PGE_2_ + muscimol^24*h*^ group (administered NS/muscimol ipsilaterally 24 h after the 2nd injection before MWT assessment). In addition, to test whether the Car/PGE_2_ injection change the function of GABAAR, the rats were randomly divided into the (1) the Car + PGE_2_ + NS^24*h*^ group and (2) Car + PGE_2_ + Pic^24*h*^ group or (1) the Car + PGE_2_ + NS^24*h*^ group and (2) Car + PGE_2_ + Mus^24*h*^ group. Then, in order to test whether GABAAR was involved in the Car/PGE_2_ injection induced long-lasting hyperalgesia, the rats were randomly divided into (1) the Car + PGE_2_ + NS group and (2) the Car + PGE_2_ + Mus group (administered NS/muscimol ipsilaterally 10 min before the 2nd injection) or (1) the NS + PGE_2_ group, (2) Car + PGE_2_ group and (3) Car + Pic group (administered picrotoxin instead of PGE_2_). In addition, to test whether the GABABR was involved in the long-lasting hyperalgesia induced by Car/PGE_2_ injection, GABABR was selectively activated 24 h after PGE_2_ injection before MWT assessment by baclofen, a selective GABABR agonist. The rats were randomly divided into (1) the Car + PGE_2_ group and (2) the Car + PGE_2_ + Bac^24*h*^ group.

In step two, we tried to clarify the relationship between GABAAR and PKCε in the DRG because PKCε is one of the most important molecular markers of hyperalgesic priming in male rats (Joseph et al., 2003). First, we showed that PKCε was activated by Car/PGE_2_ injection, as in previous studies. In this part, rats in the (1) the Normal group, (2) NS + PGE_2_ group and (3) Car + PGE_2_ group from step one were used. The colocalization of PKCε and GABAAR was also investigated in this part of the experiment. Then, we explored whether inhibition or activation of PKCε affects the expression of GABAAR in the L4-L6 DRGs. In this part of the experiment, the rats were divided into: (1) the Car + PGE_2_ + NS group and (2) Car + PGE_2_ + PKCεV1-2 group (administered PKCεV1-2, a selective PKCε inhibitor, ipsilaterally10 min before the 2nd injection) or (1) the NS + PGE_2_ group, (2) Car + PGE_2_ group and (3) Car + ψεRACK group (administered ψεRACK instead of PGE_2_). The expression levels of both GABAAR and PKCε in the L4-L6 DRGs were investigated. In addition, we tested whether activation or inhibition of GABAAR affects the activation of PKCε. In this part of the experiment, the rats in the (1) Car + PGE_2_ + NS group and (2) Car + PGE_2_ + Mus group or in the (1) NS + PGE_2_ group, (2) Car + PGE_2_ group and (3) Car + Pic group from step one were used. The expression level of GABAAR or PKCε in the L4-L6 DRGs was tested. Finally, we observed whether inhibition of PKCε can regulate hyperalgesic priming induced by GABAAR activation and the expression level of GABAAR in the L4-L6 DRGs. In this part of the experiment, the rats were randomly divided into (1) the Car + Pic + NS group and (2) the Car + Pic + PKCεV1-2 group (administered PKCεV1-2 10 min before picrotoxin injection). Furthermore, we observed whether activation of GABAAR regulates hyperalgesic priming induced by PKCε activation and the expression level of PKCε in the L4-L6 DRGs. In this part of the experiment, the rats were randomly divided into (1) the Car + RACK + NS group and(2) the Car + RACK + Mus group (administered muscimol 10 min before ψεRACK injection).

In step three, we explored the effect of EA on hyperalgesic priming and whether GABAAR is involved in this effect. In this part of the experiment, the rats were randomly divided into four groups: (1) the NS + PGE_2_ group, (2) Car + PGE_2_ group, (3) Car + PGE_2_ + EA group, and (4) Car + PGE_2_ + sham EA group. Then, we tried to block the effect of EA on hyperalgesic priming by using picrotoxin and assessed the expression of PKCε and GABAAR. In this part of the experiment, the rats were randomly divided into three groups: (1) the NS + PGE_2_ group, (2) Car + PGE_2_ group, and (3) Car + PGE_2_ + EA + Pic group (injected with picrotoxin before EA intervention each time after the 2nd injection).

### Statistical Analysis

All data are presented as the mean ± standard error of the mean (x̄ ± SEM). Student’s *t*-test was used to compare two independent samples, whereas analysis of variance (ANOVA) followed by Bonferroni’s multiple comparison tests was used to compare three or more samples. *P* < 0.05 was considered statistically significant.

## Results

### Low Expression of GABAAR in the L4–L6 DRGs in the Hyperalgesic Priming Model

**FIGURE 1:**
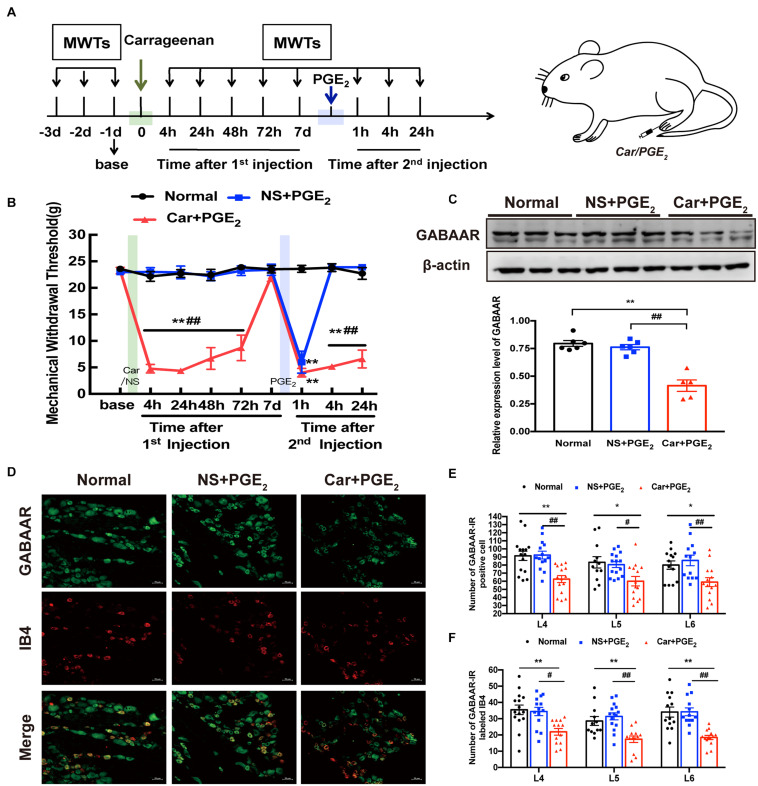
GABAAR in the L4-L6 DRGs plays a key role in the hyperalgesic priming model **(A)** Schedule of the experimental protocol. The hyperalgesic priming model was established by sequential intraplantar injection of carrageenan (Green arrow, 1 mg/100 μL/paw) and PGE_2_ (Blue arrow, 100 ng/25 μL/paw) into the left hind paw. **(B)** The mechanical withdrawal threshold (MWT) of rats that received carrageenan (Green line) and PGE_2_ (Blue line) injections. **(C)** The quantification of the Western blots results and a representative Western blot showing GABAAR protein isolated from the DRG 24 h after 2nd injection. **(D)** Representative images of GABAAR-IR positive neurons (green) merged with IB4 neurons (red) in lumbar DRG of hyperalgesic priming rats. **(E)** The quantification of GABAAR-IR positive neurons in L4, L5, and L6 DRG, respectively. **(F)** The quantification of GABAAR-IR positive neurons labeled with IB4 in L4, L5, and L6 DRG, respectively. 15 sections from three different rats. Data are presented as mean ± SEM, *n* = 6; *P < 0.05, **P < 0.01 vs. Normal group, ^#^*P* < 0.05, ^##^*P* < 0.01 vs. NS + PGE_2_ group. *P* < 0.01 indicate significant differences according to ANOVA followed by Bonferroni *post hoc* analysis.

A hyperalgesic priming model was established by sequentially injecting Car/PGE_2_ into the left hind paws of rats. The experiment was conducted according to the schedule shown in Figure 1A. MWT were assessed 1 day before the 1st injection (green arrow); 4, 24, 48, 72 h and 7 days after the 1st injection; and 1, 4, and 24 h after the 2nd injection (blue arrow). The results are shown in Figure 1B. There was no difference in basic pain threshold between the groups (*P* > 0.05). Compared with that of the Normal group and NS + PGE_2_ group, the MWT of the Car + PGE_2_ group was significantly decreased 4, 24, 48, and 72 h after the 1st injection (Figure 1B, *P* < 0.01). Subsequently, the MWT of the Car + PGE_2_ group gradually returned to baseline by 7 days after the 1st injection (*P* > 0.05). Injection of a low dose of PGE_2_, which caused only brief hyperalgesia (lasting less than 4 h) in the NS + PGE_2_ group, evoked hyperalgesia lasting at least 24 h in the Car + PGE_2_ group (Figure 1B, *P* < 0.01). All of the above results indicated that the hyperalgesic priming model was successfully established.

Then, western blotting and immunofluorescence were used to investigate the expression level of GABAAR in the L4-L6 DRGs of hyperalgesic priming model rats. The western blotting results showed that the expression level of GABAAR in the ipsilateral L4-L6 DRGs in the Car + PGE_2_ group was lower than that in the Normal and NS + PGE_2_ groups (Figure 1C, *P* < 0.01) and that the expression level of GABAAR in the NS + PGE_2_ group was not different from that in the Normal group (*P* > 0.05). Furthermore, the immunofluorescence results showed that the number of GABAAR-IR-positive cells in the L4-L6 DRGs in the Car + PGE_2_ group was lower than that in the Normal group and NS + PGE_2_ group (Figures 1D,E, *P* < 0.05). A previous study demonstrated that isolectin B4 (IB4)-positive neurons play a pivotal role in pain transition (Joseph and Levine, 2010). We further assessed the colocalization of IB4 and GABAAR. As shown in Figure 1D, IB4 and GABAAR were colocalized in the L4-L6 DRGs in the Normal, NS + PGE_2_ and Car + PGE_2_ groups. Nevertheless, PGE_2_ injection following Car administration, but not NS/PGE_2_ injection, significantly decreased the number of colabeled neurons in the L4-L6 DRGs 24 h after 2nd injection (Figures 1D,F, *P* < 0.05).

### Function of GABAAR in the L4-L6 DRGs Is Not Changed in Hyperalgesic Priming Model

Previous studies have demonstrated that changes in both GABAAR function and expression are involved in hyperalgesia (Ran et al., 2014; Obradovic et al., 2015). Especially, pervious reports indicated that PGE_2_ can modify the GABA-activated current (Yang et al., 2015). So, we first to test whether PGE_2_ or Car/PGE_2_ injection would change the function of GABAAR activation from analgesia to hyperalgesia.

**FIGURE 2:**
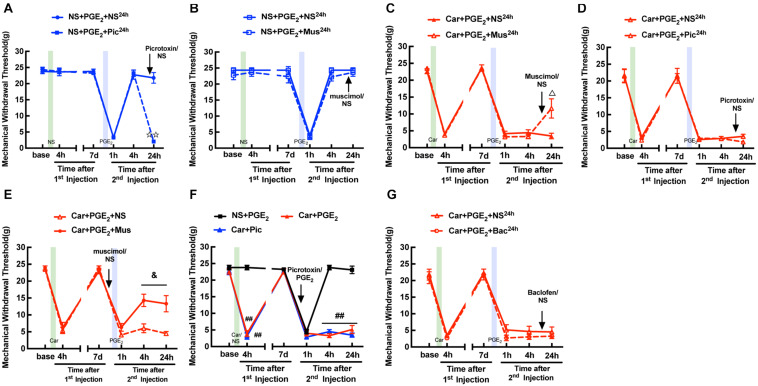
Function of GABAAR in the L4-L6 DRGs is not changed in hyperalgesic priming model. **(A)** Picrotoxin (1 μM/25 μL/paw) injection at 24 h after the 2nd injection significantly decreased the MWT of sham hyperalgesic priming rats. **(B)** Muscimol (10 μM/25 μL/paw) injection at 24 h after the 2nd injection produced little effect on MWT of sham hyperalgesic priming rats. **(C)** Muscimol injection at 24 h after 2nd injection significantly increased the MWT of hyperalgesic priming rats. **(D)** Picrotoxin injection at 24 h after the 2nd injection produced little effect on MWT of hyperalgesic priming rats. **(E)** MWT of hyperalgesic priming rats that received Muscimol injection 10 min before the PGE_2_ injection. **(F)** MWT of rats that received Picrotoxin injection following by carrageenan. **(G)** Baclofen (60 μg/25 μL/paw) injection at 24 h after the 2nd injection failed to affect the MWT of hyperalgesic priming rats. All Data are presented as mean ± SEM, *n* = 6 rats per group; 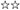 *P* < 0.01 vs. NS + PGE_2_ + NS^24*h*^ group, ^Δ^ *P* < 0.05 vs. Car + PGE_2_ + NS^24*h*^ group, ^&^*P* < 0.05 vs. Car + PGE_2_ + NS group, ^##^*P* < 0.01 vs. NS + PGE_2_ group. Student’s *t*-test was used to compare two independent samples; Three independent samples are according to ANOVA followed by Bonferroni *post hoc* analysis.

Blocking GABAAR with picrotoxin (a selective GABAAR antagonist) decreased the MWT of rats treated with NS/PGE_2_ injection 24 h after then returned to baseline levels (Figure 2A, *P* < 0.01). And muscimol (a special GABAAR agonist) did not affect the MWT of rats treated with NS/PGE_2_ injection at the same time (Figure 2B, *P* > 0.05). In addition, muscimol significantly reversed the hyperalgesia induced by Car/PGE_2_ injection 24 h after the 2nd injection (Figure 2C, *P* < 0.05). And picrotoxin injection failed to reverse the decrease in the MWT of hyperalgesic priming model rats at the same time (Figure 2D, *P* > 0.05). All of above results indicated that peripheral injection of PGE_2_, either after NS or Car injection, did not change the function of GABAAR. Furthermore, results also indicated that the change of GABAAR expression in DRG was involved in the maintenance of the hyperalgesia induced by Car/PGE_2_.

Then, we further tested whether GABAAR activation can regulate the hyperalgesic priming induced by Car/PGE_2_ injection. Muscimol was intraplantarly injected into the ipsilateral paw 10 min before PGE_2_ injection. The results revealed that the MWT of the Car + PGE_2_ + Mus group was significantly increased at 4 and 24 h after the 2nd injection (Figure 2E, *P* < 0.05). Furthermore, injection of picrotoxin, like that of PGE_2_, produced a long-lasting decrease in the MWT (Figure 2F, *P* < 0.01). All of these results indicated that GABAAR is involved in the initiation of hyperalgesic priming.

To further confirm the role of GABAAR, but not GABABR in pain modulation, we attempted to alter the MWT of hyperalgesic priming model rats with baclofen (a selective GABABR agonist). As shown in Figure 2G, baclofen had little effect on the pain thresholds of the hyperalgesic priming model rats (Figure 2G, *P* > 0.05). All these results indicate that GABAAR, but not GABABR may be involved in the maintenance of hyperalgesic priming.

### A Change in GABAAR Expression Is Involved in PKCε Activation

**FIGURE 3:**
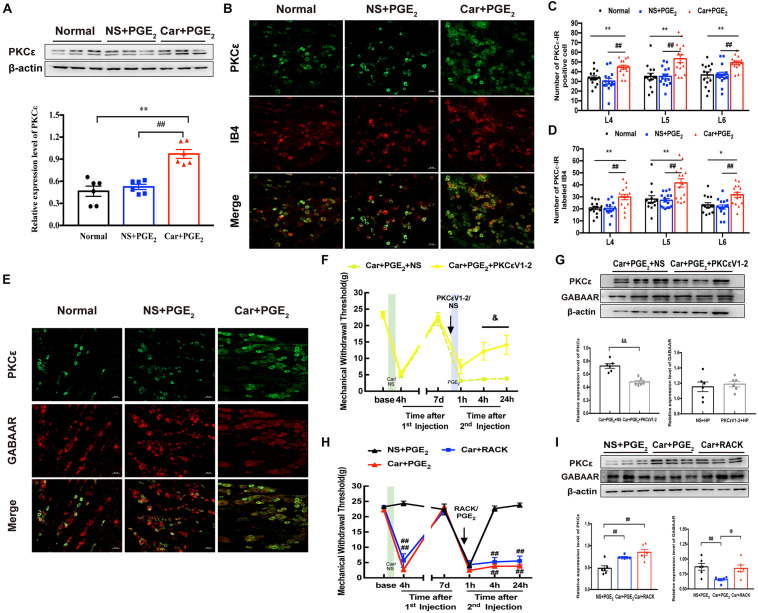
PKCε contributes to the pain transition, but not regulates GABAAR expression. **(A)** The quantification of the Western blots results and a representative Western blot showing PKCε protein isolated from the DRG 24 h after 2nd injection. **(B)** Representative images of PKCε-IR positive neurons (green) merged with IB4 neurons (red) in lumbar DRG of hyperalgesic priming rats. **(C)** The quantification of PKCε-IR positive neurons in L4, L5, and L6 DRG, respectively. **(D)** The quantification of PKCε-IR positive neurons labeled with IB4 in L4, L5, and L6 DRG, respectively. 15 sections from three different rats. **(E)** Representative images of PKCε-IR positive neurons (green) merged with GABAAR-IR positive neurons (red) in lumbar DRG of hyperalgesic priming rats. **(F)** MWT of hyperalgesic priming rats that received PKCεV1-2 (1 μg/25 μL/paw) injection 10 min before the PGE_2_ injection. *n* = 6. **(G)** The quantification of the Western blots results and a representative Western blot showing PKCε and GABAAR protein isolated from the L4-L6 DRG 24 h after 2nd injection. **(H)** MWT of rats that received ψεRACK (1 μg/25 μL/paw) injection following by carrageenan. *n* = 6. **(I)** The quantification of the Western blots results and a representative Western blot showing PKCε and GABAAR protein isolated from the L4-L6 DRG 24 h after 2nd injection. Data are presented as mean ± SEM, *n* = 6; *P < 0.05, **P < 0.01 vs. Normal group, ^##^*P* < 0.01 vs. NS + PGE_2_ group. ^&^*P* < 0.05, ^&⁣&^*P* < 0.01 vs. Car + PGE_2_ + NS group, Φ*P* < 0.05 vs. Car + PGE_2_ group. Student’s *t*-test was used to compare two independent samples; Three independent samples are according to ANOVA followed by Bonferroni *post hoc* analysis.

It is generally believed that the activation of PKCε in the DRG contributes to the transition from acute to chronic pain (Ferrari et al., 2013; Zha et al., 2015). Here, we investigated the expression of PKCε in the L4-L6 DRGs by immunofluorescence and western blotting at 24 h after the 2nd injection. As expected, the western blotting results indicated that PKCε expression in the L4-L6 DRGs was higher in rats in the Car + PGE_2_ group than rats in the Normal and NS + PGE_2_ group (Figure 3A, *P* < 0.01). Consistent with the western blotting results, the number of PKCε-IR neurons in the L4-L6 DRGs in the Car + PGE_2_ group was much higher than that in the Normal and NS + PGE_2_ groups (Figures 3B,C, *P* < 0.01). We also measured the expression of PKCε in IB4-positive neurons. The results showed that were more cells in which PKCε and IB4 were colocalized in Car + PGE_2_ group than in the Normal and NS + PGE_2_ groups (Figures 3B,D, *P* < 0.05). All these results are consistent with previous reports and suggest that PKCε is activated in hyperalgesia priming model rats.

Then, we investigated whether PKCε regulates the expression of GABAAR in the DRG, as previous studies indicated that PKCε can regulate GABAAR expression (Li et al., 2015; Puia et al., 2015). As shown in Figure 3E, colocalization of GABAAR and PKCε was observed in all three groups, which suggests that there may be a relationship between GABAAR and PKCε expression. PKCεV1-2 (a specific PKCε antagonist) was intraplantarly injected into the ipsilateral paws of hyperalgesic priming model rats 10 min before the 2nd injection. PKCεV1-2 significantly inhibited the pain transition induced by Car/PGE_2_ injection (Figure 3F, *P* < 0.05), which is consistent with previous studies (Wang et al., 2020). However, it failed to regulate the expression of GABAAR in the ipsilateral L4-L6 DRGs (Figure 3G, *P* > 0.05) but successfully inhibited the expression of PKCε (Figure 3G, *P* < 0.01). ψεRACK (a selective agonist peptide of PKCε) was also used to investigate the relationship between GABAAR and PKCε. Injection of ψεRACK but not PGE_2_ after Car injection induced hyperalgesic priming, significantly decreased the MWT of rats (Figure 3H, *P* < 0.01), and significantly activated PKCε expression in the L4-L6 DRGs (Figure 3I, *P* < 0.01). However, it did not affect the expression level of GABAAR in the ipsilateral L4-L6 DRGs (Figure 3I, *P* > 0.05).

**FIGURE 4:**
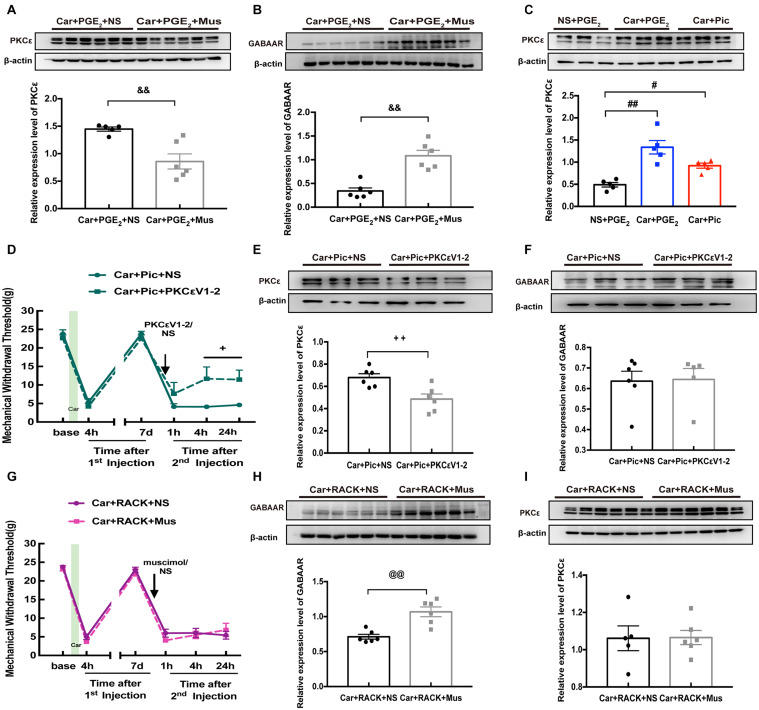
The relationship between GABAAR and PKCε in L4-6 DRGs of hyperalgesic priming rats. The quantification of the Western blots results and a representative Western blot showing PKCε **(A)** and GABAAR **(B)** protein isolated from the L4-L6 DRG 24 h after 2nd injection. The Muscimol (10 μM/25 μL/paw) was administrated 10 min before PGE_2_ injection. **(C)** The quantification of the Western blots results and a representative Western blot showing PKCε protein isolated from the DRG 24 h after 2nd injection. The Picrotoxin (1 μM/25 μL/paw) was administrated instead of PGE_2_. **(D)** Injection of PKCεV1-2 (1 μg/25 μL/paw) reversed the long-lasting hyperalgesia induced by Picrotoxin injection following by carrageenan. The quantification of the Western blots results and a representative Western blot showing PKCε **(E)** and GABAAR **(F)** protein isolated from the DRG 24 h after 2nd injection. **(G)** Muscimol injected 10 min before 2nd injection failed to regulate the hyperalgesic priming produced by ψεRACK (1 μg/25 μL/paw) injection following carrageenan. The quantification of the Western blots results and a representative Western blot showing PKCε **(H)** and GABAAR **(I)** protein isolated from the DRG 24 h after 2nd injection. Data are presented as mean ± SEM, *n* = 6; ^&⁣&^*P* < 0.01 vs. Car + PGE_2_ + NS group; ^#^*P* < 0.05, ^##^*P* < 0.01 vs. NS + PGE_2_ group; ^+^*P* < 0.05, ^++^*P* < 0.01 vs. Car + Pic + NS group; ^@@^*P* < 0.01 vs. Car + PGE_2_ + NS group. Student’s *t*-test was used to compare two independent samples; Three independent samples are according to ANOVA followed by Bonferroni *post hoc* analysis.

All the above results indicate that GABAAR may not be regulated by PKCε activation or that GABAAR may regulate PKCε activation. As shown in Figure 2E, intraplantar injection of muscimol significantly reversed the induction of hyperalgesia by PGE_2_ injection following carrageenan. Therefore, we further observed whether muscimol regulates the expression levels of PKCε and GABAAR in the ipsilateral L4-L6 DRGs. The western blotting results showed that muscimol significantly downregulated the expression of PKCε in the ipsilateral L4-L6 DRGs 24 h after the 2nd injection (Figure 4A, *P* < 0.01) and upregulated the expression of GABAAR (Figure 4B, *P* < 0.01). These results indicate that changes in GABAAR expression in the L4-L6 DRGs may not only be involved in hyperalgesic priming but also contribute to PKCε activation. Furthermore, injection of picrotoxin rather than PGE_2_ after carrageenan injection induced a persistent decrease in the MWT of rats (Figure 2F) and increased PKCε expression in the L4-L6 DRGs (Figure 4C, *P* < 0.05). All the results show that GABAAR regulates PKCε activation in the DRGs of hyperalgesic priming model rats.

We further confirmed the relationship between PKCε activation and GABAAR expression in the hyperalgesic priming model by pharmacological methods. Analysis of MWT showed that PKCεV1-2 intraplantar administration significantly relieved the hyperalgesia caused by picrotoxin injection following carrageenan administration (Figure 4D, *P* < 0.05). In addition, the expression of PKCε in the L4-L6 DRGs was partially but significantly decreased by PKCεV1-2 injection (Figure 4E, *P* < 0.01), while PKCεV1-2 was not observed to have an effect on the expression of GABAAR (Figure 4F, *P* > 0.05). In addition, muscimol had little effect on the hyperalgesia caused by ψεRACK injection following carrageenan administration (Figure 4G, *P* > 0.05) but reversed the long-lasting pain caused by PGE_2_ injection following carrageenan administration (Figure 2E). Moreover, although muscimol injection significantly upregulated the expression of GABAAR in the L4-L6 DRGs (Figure 4H, *P* < 0.01), it did not affect the activating effect of ψεRAKC on PKCε (Figure 4I, *P* > 0.05). All these results indicate that lower expression of GABAAR is responsible for the activation of PKCε in the L4-L6 DRGs.

### EA Reversed the Transition of Pain by Regulating GABAAR Expression

**FIGURE 5:**
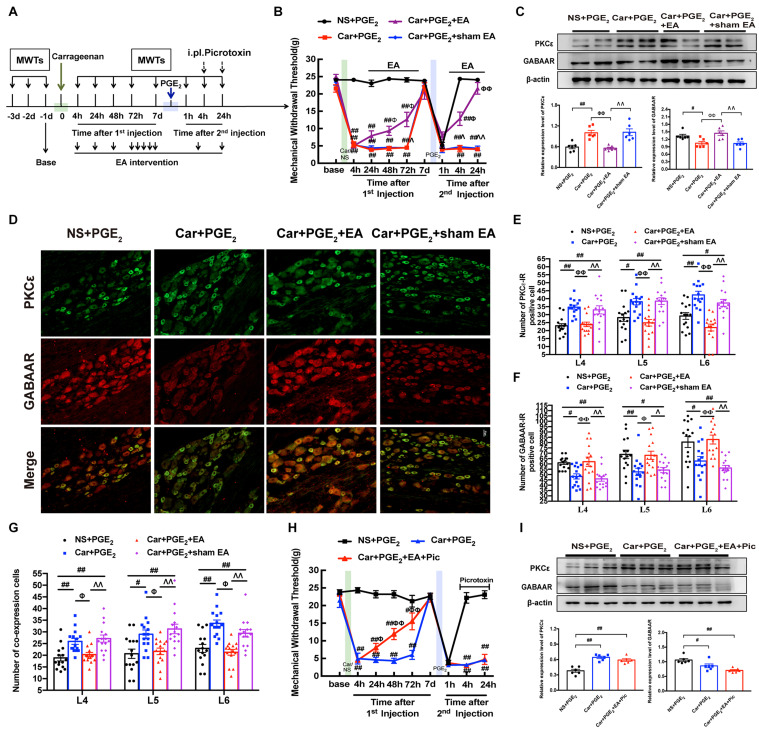
EA regulates the transition from acute to chronic pain and GABAAR expression in the L4-L6 DRG. **(A)** The protocol of EA administration experiment. **(B)** MWT of hyperalgesic priming rats received EA or sham EA stimulation. *n* = 6. **(C)** The quantification of the Western blots results and a representative Western blot showing PKCε and GABAAR protein isolated from the DRG 24 h after 2nd injection. **(D)** Representative images of PKCε-IR positive neurons (green) merged with GABAAR-IR positive neurons (red) in lumbar DRGs of hyperalgesic priming rats. **(E)** The quantification of PKCε-IR positive neurons in L4, L5 and L6 DRG, respectively. **(F)** The quantification of GABAAR-IR positive neurons in L4, L5 and L6 DRG, respectively. **(G)** The quantification of PKCε-IR positive neurons labeled with GABAAR in L4, L5 and L6 DRG, respectively. 15 section from three different rats. **(H)** Picrotoxin (1 μM/25 μL/paw) injection blocked the analgesic effect of EA on hyperalgesic priming rats. *n* = 6. **(I)** The quantification of the Western blots results and a representative Western blot showing PKCε and GABAAR protein isolated from the DRG 24 h after 2nd injection. Data are presented as mean ± SEM, *n* = 6; ^##^*P* < 0.01 vs. NS + PGE_2_ group; ^ΦΦ^ *P* < 0.01 vs. Car + PGE_2_ group; ^ΛΛ^ *P* < 0.01 vs. Car + PGE_2_ + EA group. *P* < 0.01 indicate significant differences according to ANOVA followed by Bonferroni *post hoc* analysis.

We further investigated EA’s regulatory effect on pain transition and the underlying mechanism. The EA stimulation procedure is shown in Figure 5A. As shown in Figure 5B, EA significantly promoted the reduction in MWT induced by carrageenan injection from 48 h to 72 h after the 1st injection (*P* < 0.01), and significantly increased the MWT 4 and 24 h after the 2nd injection (*P* < 0.01). Sham EA had little effect on the MWT of hyperalgesic priming model rats (*P* > 0.05).

Consistent with the results shown in Figures 1C, 3A, PGE_2_ injection following carrageenan administration upregulated PKCε expression in the ipsilateral L4-L6 DRGs of hyperalgesic priming model rats and downregulated GABAAR expression (Figure 5C, *P* < 0.05). EA stimulation significantly inhibited the overexpression of PKCε in the L4-L6 DRGs and promoted the expression of GABAAR (Figure 5C, *P* < 0.01). In contrast, sham EA had no such effect (*P* > 0.05).

Consistent with the above results, the number of PKCε-IR-positive cells was increased in the L4-L6 DRGs of hyperalgesic priming model rats (Figures 5D,E, *P* < 0.05), and the number of GABAAR-IR-positive cells was decreased (Figures 5D,F, *P* < 0.05). In contrast, EA stimulation significantly reduced the number of PKCε-IR-positive cells in the L4-L6 DRGs (Figures 5D,E, *P* < 0.01). Moreover, EA stimulation significantly increased the number of GABAAR-positive cells in the L4-L6 DRGs in the Car + PGE_2_ + EA group compared with the Car + PGE_2_ group (Figures 5D,F, *P* < 0.05). We also tested the colocalization of PKCε and GABAAR in the L4-L6 DRGs. PGE_2_ injection following carrageenan administration upregulated the number of colabeled cells in the L4-L6 DRGs 24 h after 2nd injection (Figure 5G, *P* < 0.05). EA significantly downregulated the number of colabeled cells in the L4-L6 DRGs at the same time (Figure 5G, *P* < 0.05). All of these results indicate that EA may regulate pain transition and PKCε by promoting GABAAR expression in the L4-L6 DRGs.

To investigate whether GABAAR is involved in the effect of EA against pain transition, picrotoxin was injected 10 min before each EA intervention after the 2nd injection (Figure 5A), and the changes in the MWT in rats from each group were observed. Consistent with the findings shown in Figure 5B, EA efficiently alleviated the mechanical allodynia induced by carrageenan injection from 24 h to 72 h after the 1st injection (Figure 5H, *P* < 0.05). However, picrotoxin blocked the analgesic effect of EA on hyperalgesic priming model rats, as the MWT of the Car + PGE_2_ + EA + Pic group was not different from that of the Car + PGE_2_ group (Figure 5H, *P* > 0.05). Furthermore, picrotoxin abolished the regulatory effect of EA on PKCε activation in the lumbar DRG, as shown in Figure 5I. There was no significant difference between the Car + PGE_2_ group and Car + PGE_2_ + EA + Pic group in the protein expression level of PKCε (Figure 5I, *P* > 0.05), but the expression level of PKCε in the L4-L6 DRGs in the NS + PGE_2_ group was much lower than that in the Car + PGE_2_ group (Figure 5I, *P* < 0.01). Furthermore, picrotoxin blocked EA’s regulatory effect on GABAAR expression (Figure 5I, *P* > 0.05), as the expression level of GABAAR in the Car + PGE_2_ + EA + Pic group was not different from that in the Car + PGE_2_ group (Figure 5I, *P* > 0.05) and was significantly lower than that in the NS + PGE_2_ group (Figure 5I, *P* < 0.01).

## Discussion

Hyperalgesic priming is a model used to investigate the mechanism of pain transition. In the model, PGE_2_ get the ability to produce a long-lasting hyperalgesia due to the pre-exposure of peripheral nerve terminal to the inflammation (Kim et al., 2015; Megat et al., 2018). Previous studies demonstrated the PKCε activation in the DRG is the main reason for the long-lasting hyperalgesia (Ferrari et al., 2014; Kandasamy and Price, 2015). In addition, the estrogen can prevent the hyperalgesia produced by the PKCε activation, which indicated that the PKCε activation can only induce the hyperalgesic priming in the male mammals (Joseph et al., 2003). So, only the male rats were used in this study.

GABAAR is one of the most important inhibitory receptors, is widely distributed in the peripheral and central nervous systems (Bhisitkul et al., 1987), and has a clear inhibitory effect on the excitability of neurons (Witschi et al., 2011). Although GABAAR is widely distributed from primary afferent nerve terminals to axons and a growing body of data suggests that it is involved in pathological pain, the contribution of GABAAR to chronic pain is still highly controversial (Wilke et al., 2020). It is generally believed that GABAAR located at the central terminals of primary afferents in the spinal dorsal horn exerts antinociceptive effects through presynaptic inhibition, which means that depolarization caused by GABAAR in the dorsal horn alleviates pain. However, how GABAAR expressed in the DRG modulates the excitation of primary afferent neurons and is involved in hypersensitization is still unclear. In the current study, we firstly investigated the expression level of GABAAR in the L4-L6 DRGs. The western blotting and immunofluorescence results both indicated a decrease in GABAAR expression in the lumbar DRG of the hyperalgesic priming model rats. In addition, GABAAR expression in the lumbar DRG was restored with relief of hyperalgesic priming. All of these results suggest that GABAAR is involved in pain transition.

Although it was well established in the 1970s that primary sensory neurons respond to GABA stimulation (De Groat et al., 1972), whether GABAAR exerts an antihyperalgesia effect through depolarization or hyperpolarization is unclear. A multitude of studies has shown that peripheral administration of muscimol evokes nocifensive behaviors, whereas selective GABAAR blockers inhibit pain-like behaviors (Bravo-Hernández et al., 2014; Jang et al., 2017). However, there is also evidence demonstrating that GABAAR activation can alleviate pain-like behaviors and that picrotoxin causes hypersensitivity (Naik et al., 2008, 2012; Ran et al., 2014). Furthermore, some reports have indicated that peripheral inflammation and nerve injury strongly affect the function and expression of GABAAR (Chen et al., 2014; Chakrabarti et al., 2018). In the current study, muscimol significantly alleviated the long-lasting hyperalgesia evoked by PGE_2_ injection following Car administration and did not cause pain-like behaviors after PGE_2_ injection. Furthermore, picrotoxin significantly decreased the pain threshold of sham hyperalgesic priming model rats and failed to alleviate chronic pain in hyperalgesic priming model rats. All of this evidence indicates that GABAAR activation can produce an analgesic effect on the rats either after PGE_2_ or Car/PGE_2_ injection. Furthermore, activation of GABAAR produces an analgesic effect on hyperalgesic priming model mammals 24 h after PGE_2_ injection, which indicated that the GABAAR may be involved in the prolonged phase of the PGE_2_-induced hyperalgesia. According to the results, we further investigated whether activation of GABAAR can regulate the transition from acute to chronic pain. As expected, pharmacological activation of GABAAR before PGE_2_ injection regulated the induction of priming by PGE_2_ injection following Car administration. Moreover, pharmacological blockade of GABAAR with the specific antagonist picrotoxin mimicked hyperalgesic priming in rats. All of these results suggest that GABAAR is involved in the initial phase of pain transition.

It has been reported that hyperalgesia, the duration of which is short, induced by PGE_2_ is dependent on PKA in unprimed rats (Aley and Levine, 1999), but is PKCε-dependent in the primed state, starting 1 h after the injection of PGE_2_ (Parada et al., 2003; Zha et al., 2015). It is now recognized that the activation of PKCε in DRG neurons increases the excitability of neurons and induces pain transition. Inhibiting the activation of PKCε can effectively prevent the prolonged mechanical hyperalgesia induced by PGE_2_ in a carrageenan-induced priming model (Parada et al., 2003; Araldi et al., 2019). Consistent with previous studies, our results showed that PKCε activation in the L4-L6 DRGs is necessary for pain transition. However, little is known about whether there is an interaction between GABAAR and PKCε in the transition from acute to chronic pain. A series of pharmacological experiments were used to verify the link between GABAAR and PKCε in the DRGs of hyperalgesic priming model rats in the current study. Our results suggested that GABAAR and PKCε are coexpressed in the same DRG neurons. In addition, picrotoxin not only mimicked hyperalgesic priming in rats but also activated PKCε in the L4-L6 DRGs. More importantly, muscimol had no effect on the MWT of hyperalgesic priming model rats or PKCε expression in the L4-L6 DRGs induced by ψεRACK but significantly reversed the decreases in the MWT of hyperalgesic priming model rats and PKCε expression in the L4-L6 DRGs induced by PGE_2_. The other results were consistent: preinjection of PKCεV1-2 had little effect on the expression of GABAARin the L4-L6 DRGs but prevented the induction of pain transition. All of these results suggest that GABAAR contributes to hyperalgesic priming by activating PKCε.

Clinically, chronic pain remains a serious public health problem and is one of the costliest conditions in the field of health care (Gaskin and Richard, 2012). EA is a form of acupuncture and is widely used to treat various types of pain (Fang et al., 2018; Ali et al., 2020). Previous studies have proven that EA can prevent the induction of pain transition and that its effect is most likely achieved through a PKCε-related pathway (Wang et al., 2020). Previous studies have indicated that upregulation of the expression of GABAAR in the central nervous system is related to the analgesic effect of EA (Jiang et al., 2018; Zheng et al., 2020). However, there are few reports on whether EA regulates GABAAR expression or function in the peripheral nervous system. Here, we observed that EA treatment significantly prevented the prolonged hyperalgesia induced by PGE_2_ injection following Car and decreased the expression of PKCε in the L4-L6 DRGs of hyperalgesic priming model rats, which is consistent with our previous report (Wang et al., 2020). Furthermore, EA upregulated the expression of GABAAR in the L4-L6 DRGs of hyperalgesic priming model rats. All of these results indicate that EA may alleviate hyperalgesic priming by upregulating GABAAR expression in the lumbar DRG. According to the results, picrotoxin was used to reverse the analgesic effect of EA on hyperalgesic priming. The pain behavior test and western blotting suggested that picrotoxin abolished both the regulatory effect of EA on the pain threshold and PKCε activation. All of the above results suggest that EA’s regulatory effect on hyperalgesic priming and PKCε activation is positively related to its ability to upregulate GABAAR expression in the DRG.

## Conclusion

In conclusion, the results of this study suggested that changes in GABAAR expression in the L4-L6 DRGs contribute to hyperalgesic priming type I. EA regulates this type of pain transition by promoting GABAAR expression in the peripheral nervous system.

## Data Availability Statement

The original contributions presented in the study are included in the article/supplementary material, further inquiries can be directed to the corresponding author/s.

## Ethics Statement

The animal study was reviewed and approved by Animal Care and Welfare Committee of Zhejiang Chinese Medical University, Zhejiang, China.

## Author Contributions

SW and JD performed the data analysis and manuscript writing. DX performed the EA administration. FS and MQ performed the paw with draw threshold testing. SW, DX, and JD performed the western blotting and immunofluorescence. XS, YL, XJ, and BL performed the revising. JiF and JuF designed the experimental protocols. All authors agreed to be accountable for all aspects of the work.

## Conflict of Interest

The authors declare that the research was conducted in the absence of any commercial or financial relationships that could be construed as a potential conflict of interest.
